# Peripheral immunophenotype in dementia with Lewy bodies and Alzheimer’s disease: an observational clinical study

**DOI:** 10.1136/jnnp-2020-323603

**Published:** 2020-09-23

**Authors:** Jay Amin, Delphine Boche, Zoe Clough, Jessica Teeling, Anthony Williams, Yifang Gao, Lindsey Chudley, Laurie Lau, Florence Smith, Scott Harris, Clive Holmes

**Affiliations:** 1 Clinical Neurosciences, Clinical and Experimental Sciences, Faculty of Medicine, University of Southampton, Southampton, UK; 2 Memory Assessment and Research Centre, Southern Health NHS Foundation Trust, Southampton, UK; 3 Faculty of Natural and Environmental Science, University of Southampton, Southampton, UK; 4 Regional Clinical Immunology Laboratory, Cancer Sciences, Faculty of Medicine, University of Southampton, Southampton, UK; 5 Medical Statistics, School of Primary Care, Population Sciences and Medical Education, Faculty of Medicine, University of Southampton, Southampton, UK

## Abstract

**Background:**

Inflammation plays a key role in the aetiology and progression of Alzheimer’s disease (AD). However, the immunophenotype of the second most common neurodegenerative cause of dementia, dementia with Lewy bodies (DLB), remains unclear. To date there have been no studies examining peripheral inflammation in DLB using multiplex immunoassay and flow cytometry concomitantly. We hypothesised that, using blood biomarkers, DLB would show an increased proinflammatory profile compared with controls, and that there would be a distinct profile compared with AD.

**Methods:**

93 participants (31 with DLB, 31 with AD and 31 healthy older controls) completed a single study visit for neuropsychiatric testing and phlebotomy. Peripheral blood mononuclear cells were quantified for T and B cell subsets using flow cytometry, and serum cytokine concentrations were measured using multiplex immunoassay.

**Results:**

We detected reduced relative numbers of helper T cells and reduced activation of B cells in DLB compared with AD. Additionally, interleukin (IL)-1β was detected more frequently in DLB and the serum concentration of IL-6 was increased compared with controls.

**Conclusions:**

Peripheral inflammation is altered in DLB compared with AD, with T cell subset analysis supporting a possible shift towards senescence of the adaptive immune system in DLB. Furthermore, there is a proinflammatory signature of serum cytokines in DLB. Identification of this unique peripheral immunophenotype in DLB could guide development of an immune-based biomarker and direct future work exploring potential immune modulation as a novel treatment.

## Introduction

There are an estimated 35.6 million people living with dementia worldwide,^1^ yet there are still no disease-modifying treatments available to stop or reverse the causes of dementia. While interventional trials with non-steroidal anti-inflammatory drugs have proved unsuccessful in Alzheimer’s disease (AD),^2^ epidemiological studies support a role for a variety of chronic inflammatory conditions being risk factors for the disease.^3^

Dementia with Lewy bodies (DLB) is the second most common neurodegenerative cause of dementia, accounting for 4.2%–7.5% of cases.^4^ However, people with DLB often experience delays in accurate diagnosis or misdiagnosis as either AD or Parkinson’s disease (PD).^5^ The often devastating combination of fluctuating cognition, recurrent visual hallucinations and motor features of Parkinsonism mean that DLB has a poorer prognosis compared with AD, with higher healthcare costs, greater caregiver stress and increased mortality.^6 7^ Developing biomarkers to improve diagnostic accuracy in DLB, as well as opening new avenues for therapeutics, therefore warrants urgent attention.

The occurrence of acute systemic infections, chronic peripheral infections such as periodontitis, and conditions associated with chronic inflammation such as atherosclerosis and obesity, have all been implicated in the aetiology or accelerated progression of AD.^8–10^ Furthermore, genetic polymorphisms involved in inflammatory processes are known risk factors for AD.^11–13^ Inflammation thus plays a key role in the aetiology and progression of AD. The availability of more detailed examination of blood markers of inflammation in dementia has grown with the adoption of techniques such as blood cytokine analysis and flow cytometry.

Few studies have investigated peripheral cytokine concentrations in DLB, compared with AD.^14^ We previously showed that increased serum levels of proinflammatory cytokines interleukin (IL)-6 and Tumour Necrosis Factor α (TNFα) in DLB are associated with greater neuropsychiatric symptoms and worse cognition, respectively.^15^ Another study showed significantly higher plasma concentrations of IL-1β, IL-2, IL-4 and IL-10 in mild cognitive impairment-DLB (MCI-DLB), but no difference between controls and DLB.^16^ Serum concentrations of MIP-3a, IL-2 and IL-17 have been shown to be elevated, and IL-8 reduced, in DLB compared with controls, along with increased cerebral inflammation in early DLB using in-vivo Positron Emission Tomography (PET) brain imaging.^17^ In PD a meta-analysis confirmed increased blood concentrations of IL-1β, IL-2, IL-6, IL-10 and C reactive protein,^18^ while peripheral IL-1β, IL-6, IL-10 and TNFα were elevated in a review of patients with PD and PD dementia.^19^ Intriguingly, a more proinflammatory profile of peripheral cytokines predicts worse disease progression in PD.^20^

In addition to innate immune signatures, there is increasing evidence of changes to adaptive immunity in neurodegenerative diseases. Alterations in peripheral blood mononuclear cell (PBMC) subsets in AD have not yet been consistently replicated. CD4+ helper T cell subsets have been shown to be reduced in AD and PD^21 22^ with preferential depletion of naïve cells. Reduced CD8+ cytotoxic T cell numbers have been shown in AD compared with controls.^23^ However, other studies have shown no alterations in CD4+ or CD8+ T cell subsets in AD.^24 25^ B cell populations have been shown to be either reduced in AD and PD,^22 25^ or unchanged in AD.^26^ Inconsistent results in this field are likely due to differences in methodologies. To our knowledge no previous work has been published examining T and B cell subsets in DLB. Improving our knowledge of the aetiology of DLB may help to inform new immunity-related therapeutic targets and guide the development of diagnostic biomarkers.

Herein, we present the findings from an observational study, which used a clinical cross-sectional cohort of DLB and patients with AD with the aim to compare peripheral immunophenotype. We hypothesised that DLB would show a proinflammatory state compared with controls, and that the immune profile would differ from AD. We also hypothesised that associations would be detected between markers of inflammation and the clinical features of DLB.

## Methods

### Study subjects

Patients with AD or DLB were recruited from local memory clinics and the Join Dementia Research platform (JDR, https://www.joindementiaresearch.nihr.ac.uk). All participants were aged between 50 and 100 years and were proficient in English. Patients with dementia had a reliable study partner, and were required to satisfy either the National Institute of Neurological and Communicative Disorders and Stroke and the Alzheimer’s Disease and Related Disorders Association criteria for probable AD or international consensus diagnostic criteria for probable DLB.^6 27^ Healthy controls were identified from JDR or patient relations, and required a Montreal cognitive assessment (MoCA) score of ≥26 points.

Exclusion criteria included: delirium within 3 months, acute infection, current excess alcohol consumption, drug abuse, mixed dementia, any psychiatric diagnosis that could interfere with participation (eg, depression or psychosis) and use of major modifiers of the immune system (eg, oral corticosteroids or TNFα inhibitors).

### Study assessments

Participants attended a single study visit for interview, physical examination and phlebotomy. Cognition was tested using the MoCA. DLB and AD participants were further tested using the free and cued selective reminding test-immediate recall (FCSRT-IR) to assess recall, clinician assessment of fluctuation (CAF) for quantification of cognitive fluctuations, neuropsychiatric inventory (NPI) to assess for psychiatric symptoms, Cornell Scale for Depression in Dementia (CSDD) to assess mood and the Movement Disorder Society Unified Parkinson’s Disease Rating Scale (UPDRS) to quantify motor symptoms.

### Blood sample collection and PBMC isolation

Venous blood was taken at a fixed time point (10:00–12:00), to minimise the influence of diurnal cytokine variation. Whole blood was allowed to clot for 30 min at room temperature and centrifuged at 1750 g for 10 min to allow storage of aliquoted cell-free serum at minus 80°C. Blood was collected in EDTA vacutainer tubes for analysis of apolipoprotein E ε4 (APOE ε4) allele status. Blood was collected in heparinised vacutainer tubes for PBMC isolation, by density gradient separation at 950 g for 25 min on Ficoll paque-plus (ThermoFisher Scientific, Loughborough, UK). PBMC were two times washed for 8 min using phosphate buffered saline (PBS, ThermoFisher Scientific, Loughborough, UK) at 700 g. Cell viability was determined using Trypan Blue (Sigma Aldrich, Gillingham, Dorset, UK), following which PBMC were suspended in 80% foetal calf serum and 20% dimethyl sulfoxide (ThermoFisher Scientific, Loughborough, UK) at a concentration of one million cells per millilitre. Aliquots of PBMC were cryopreserved at minus 80°C before transfer to a liquid nitrogen facility for storage until batch phenotype analysis using flow cytometry.

### Cytokine analysis

Serum concentrations of IL-1β, IL-2, IL-4, IL-6, IL-8, IL-10, IL-12, IL-13, TNFα and interferon (IFN)-γ were measured by multiplex immunoassay (Meso Scale Discovery Human Pro-Inflammatory V-PLEX, Rockville, Maryland, USA). Calibrators were used as per manufacturer’s instructions. Sensitivity was indicated by the lower limit of detection (LLOD) measured in pg/mL: 0.04 for IL-1β, 0.09 for IL-2, 0.02 for IL-4, 0.06 for IL-6, 0.04 for IL-8, 0.03 for IL-10, 0.11 for IL-12, 0.24 for IL-13, 0.04 for TNFα and 0.20 for IFN-γ. All samples were analysed blind to group and in duplicate, with the mean value used for analysis. Duplicate serum cytokine concentrations below the LLOD were deemed undetectable and revalued as 0.

### Flow cytometry

Eight-colour flow cytometry was used to examine PBMC subsets by measuring relative numbers of: CD3+, CD4+ and CD8+ T lymphocytes; CD19+ B lymphocytes; CD14+ monocytes; CD45RA+ naïve T lymphocytes; CCR7+ central T lymphocytes; and HLA-DR+ activated cells.

Cryopreserved PBMC were fast thawed in a water bath at 37°C, then twice washed for 5 min at room temperature with warm complete medium at 300 g. After re-suspension in PBS, viability staining was performed using Zombie violet (Pacific blue, Biolegend, UK). PBMC were incubated with fluorochrome-conjugated antibodies against CD3 (PerCP, Becton-Dickinson), CCR7 (APC-Cy7, Biolegend, UK) and CD4 (V500 AmCyan, BD Biosciences), and CD8 (PE), CD14 (PerCP-Cy5.5), CD19 (FITC), CD45RA (APC) and human leucocyte antigen-antigen D related (HLA-DR) (PE-Cy7) (all ThermoFisher Scientific, Loughborough, UK). Data from at least 10 000 T cell events were analysed using FACSDiva software on the FACSCanto II system (BD Biosciences, San Jose, California, USA). Fluorescence minus one experiments were used as negative controls.

The gating strategy was agreed prior to data analysis. Forward scatter height versus forward scatter area (FSC-A) plots were used to exclude doublet cells. Side scatter area (SSC-A) versus cell viability marker plots were used to exclude dead cells. SSC-A versus FSC-A plots helped to identify lymphocyte and monocyte populations from granulocytes. Cell populations were defined relative to the parent population, apart from HLA-DR activation which was measured as mean fluorescent intensity (MFI) of each defined cell subset.

### Power calculation

Power was based on a study that showed increased plasma concentration of IL-1β in PD compared with controls by a ratio of 1.45, with a mean of 73.7 pg/mL (SD 16.3 pg/mL) in PD, compared with 50.8 pg/mL (SD 5.9 pg/mL) in controls.^28^ Assuming an alpha of 0.05 (two-tailed) and the higher SD, 12 participants in each group gave 90% power to show a mean difference of 22.9 pg/mL, calculated using nQuery (Statistical Solutions, Cork, Ireland). Allowance for dropout was not required due to the cross-sectional nature of the study. However, to allow for larger variation and detection of smaller differences we aimed to recruit at least 30 participants per group.

### Statistical analysis

Statistical analysis was performed using IBM SPSS statistical software (V.25.0). Demographic and clinical characteristics were compared using parametric or non-parametric tests. Groups were assessed for differences in age (one-way Analysis of Variance (ANOVA), post-hoc Tukey), gender and APOE phenotype (χ^2^), MoCA score and education (Kruskal-Wallis), neuropsychiatric test scores and disease duration (Mann-Whitney U), and frequency of cognitive enhancers, antipsychotics and anti-Parkinsonian drugs (Pearson χ^2^).

Serum cytokine concentration distributions were skewed. Group (Kruskal-Wallis) and post-hoc pairwise (Dunn-Bonferroni) differences were tested. Significant cytokine variables were log10 transformed to correct for age and gender. IL-6, IL-10 and TNFα concentrations were log10 transformed to normal distribution and linear regression used. A high number of cases showed IL-1β concentrations below the LLOD and thus it was considered a binary variable (detectable classified as present and non-detectable as absent). Group (Pearson χ^2^) and post-hoc pairwise (logistic regression) differences were tested. PBMC subsets and MFI were assessed for group differences using either ANOVA or Kruskal-Wallis tests, depending on distribution. For significant results using ANOVA, linear regression and t-tests were used to detect post-hoc significant pairwise differences adjusted for age and gender.

Within the DLB group, Spearman’s rank correlation was used to assess for associations between inflammatory markers and standardised rating scale scores representing the clinical features of the disease.

Two-tailed tests with α=0.05 determined significance, with adjustment to account for multiple testing in correlation analysis (α=0.01).

## Results

Ninety-six participants took part in our study. Of these, 32 had DLB, 32 had AD and 32 were cognitively unimpaired healthy controls. One participant originally recruited to the AD group was subsequently excluded due to the diagnosis of dementia being withdrawn by the principle investigator. Phlebotomy was unsuccessful in two participants (one control and one DLB), leaving 31 participants in each group with full clinical and cytokine data. Sufficient viable PBMC were not isolated from a further three controls, five AD cases and four DLB cases.

### Demographics and clinical characteristics

Participant characteristics are summarised in table 1. The control group (mean age 66.0±8.6 years) was younger than both dementia groups, but there was no significant difference in age between AD (mean 74.1±7.4 years) and DLB (mean 73.9±7.6 years). There was no significant difference in gender, years of education or disease duration. AD and DLB groups demonstrated mild impairment on cognitive testing (measured using MoCA) compared with controls. There was no significant difference in MoCA score, FCSRT-IR total or free recall score, CSDD score or NPI total score between DLB and AD. The DLB group scored significantly higher compared with AD in scales for fluctuations (CAF) and Parkinsonism (UPDRS motor examination and total score).

**Table 1:** Clinical characteristics

	Controls (N=31)	DLB (N=31)	AD (N=31)	Test statistic P value
Age (years±SD)	66.0±8.6	73.9±7.6*	74.1±7.4*	**10.729**† **<0.001**
Gender (male:female)	14:17	20:11	18:13	2.443‡ 0.295
Years of education (years±SD)	12.7±2.5	12.7±3.2	13.3±3.8	0.170§ 0.919
Disease duration (years±SD)	–	3.8±1.7	4.5±2.4	310.0¶ 0.260
MoCA (score ±SD)	29±1	18±6*	19±5*	**60.472**§ **<0.001**
FCSRT-IR sum total (score, LQ-UQ)	–	46 39–48	42 19.5–47	344.5¶ 0.166
FCSRT-IR free recall (score, LQ-UQ)	–	18 7–24	14 5–20.5	363.5¶ 0.278
CSDD (score, LQ-UQ)	–	2 1–4	2 1–4	481.0¶ 0.994
CAF (score, LQ-UQ)	–	4** 2–8	0 0–0	**117.5**¶ **<0.001**
UPDRS total (score, LQ-UQ)	–	31** 18–45	7 4–11	**71**¶ **<0.001**
UPDRS motor examination (score, LQ-UQ)	–	14** 8–21	2 0–5	**107.5**¶ **<0.001**
NPI total (score, LQ-UQ)	–	8 6–20	6 3–11	343.5¶ 0.053
Cholinesterase inhibitors (no of patients, %)	0	30 (96.8%)¶**	24 (77.4%)*	**66.769**‡ **<0.001**
Memantine hydrochloride (no of patients, %)	0	2 (6.5%)	4 (12.9%)	4.276‡ 0.118
Antipsychotic medications (no of patients, %)	1 (3.2%)	6 (19.3%)¶**	1 (3.2%)	**6.838**‡ **0.033**
Medications for Parkinsonism (no of patients, %)	0	8 (25.8%)¶**	0	**17.506**‡ **<0.001**

Results are presented as mean±SD or median with Lower Quartile (LQ) to Upper Quartile (UQ) below, or proportions of total number. Statistical tests are denoted with symbols.

*.ANOVA.

†χ^2^.

‡χ.Kruskal-Wallis.

§Mann-Whitney U.

¶Significantly different from controls (p<0.05).

**Significantly different from AD (p<0.05).

AD, Alzheimer’s disease; CAF, clinician assessment of fluctuation; CSDD, Cornell Scale for Depression in Dementia; DLB, dementia with Lewy bodies; FCSRT-IR, free and cued selective reminding test-immediate recall; MoCA, Montreal cognitive assessment; NPI, neuropsychiatric inventory; UPDRS, Unified Parkinson’s Disease Rating Scale.

**Table 2:** Peripheral blood mononuclear cell subsets

	Markers	Cell subset	Controls (n=28)	DLB (n=27)	AD (n=26)	Test statistic P value
CD3+	CD3+	T cells	64.2 53.4–72.0	62.6 57.2–71.3	59.1 55.1–65.2	1.287* 0.526
CD4+	CD3+ CD4+	Helper T cells	57.9±16.7	49.6±19.5^β^	61.1±14.9	**3.209**† **0.046**
	HLA-DR+ MFI	Activated helper T cells	10 246 6866–14950	10 128 7730–13491	9843 8354–12019	0.247* 0.884
	CD45RA+CCR7+	Naïve helper T cells	62.2 44.7–75.0	60.4 41.9–70.5	65.4 47.6–79.7	1.760* 0.415
	CD45RA-CCR7+	Central memory helper T cells	18.5±8.1	20.6±9.3	19.5±10.8	0.343† 0.710
	CD45RA-CCR7-	Effector memory helper T cells	12.6 6.9–24.4	11.3 8.1–22.7	12.4 6.4–19.0	1.134* 0.567
	CD45RA+CCR7-	Terminal effector helper T cells	1.8 1.1–5.6	2.5 1.3–5.6	1.8 0.8–3.7	0.716* 0.699
CD8+	CD3+CD8+	Cytotoxic T cells	29.9 22.5–35.1	40.1 26.6–53.9	25.0 20.3–37.0	5.868* 0.053
	HLA-DR+MFI	Activated cytotoxic T cells	5321 4652–6712	5530 4244–6980	5879 4281–8076	0.435* 0.804
	CD45RA+CCR7+	Naïve cytotoxic T cells	29.4 13.1–40.4	21.8 13.1–31.2	28.1 13.7–48.1	1.188* 0.552
	CD45RA-CCR7+	Central memory cytotoxic T cells	5.3 2.9–8.0	4.6 2.4–6.3	5.4 2.0–7.5	0.213* 0.899
	CD45RA-CCR7-	Effector memory cytotoxic T cells	22.4 12.0–31.6	19.0 7.2–28.2	16.7 8.8–25.4	2.042* 0.360
	CD45RA+CCR7-	Terminal effector cytotoxic T cells	42.8±20.9	50.8±20.0	45.2±20.8	1.095† 0.339
CD19+	CD3-CD19+	B cells	4.7 3.6–6.0	4.7 2.3–6.9	5.5 3.8–8.2	3.101* 0.212
	HLA-DR+MFI	Activated B cells	61861±14 832	53442±1672^β^ 9	66530±15 324	**4.786**† **0.011**
CD14+	CD3-CD14+	Monocytes	15.0 8.5–20.2	15.2 10.6–22.5	17.0 11.9–23.0	1.392* 0.499

Results are presented as mean±SD or median with LQ-UQ below, and as percentage of parent population, or (for human leucocyte antigen-antigen D related) mean fluorescent intensity in standard units. Statistical tests are denoted with symbols.

Post-hoc results: α=significantly different from controls (p<0.05), β=significantly different from AD (p<0.05).

*Kruskal-Wallis.

†Significantly different from AD (p<0.05).

‡χ^2^.

CCR7, C-C chemokine receptor type 7; CD, cluster of differentiation; HLA-DR, Human leukocyte antigen – antigen D related; MFI, mean fluorescent intensity.

No control participants were taking cognitive enhancers or anti-Parkinsonian medication. Significantly more patients with DLB and AD were taking cognitive enhancers than controls. Antipsychotic and anti-Parkinsonian medication use was more prevalent in the DLB group compared with controls and AD. Use of non-steroidal anti-inflammatory drugs were similar across groups. There was no difference in prevalence of rheumatoid arthritis, hypertension or diabetes mellitus between groups.

### PBMC subsets

**Figure 1:**
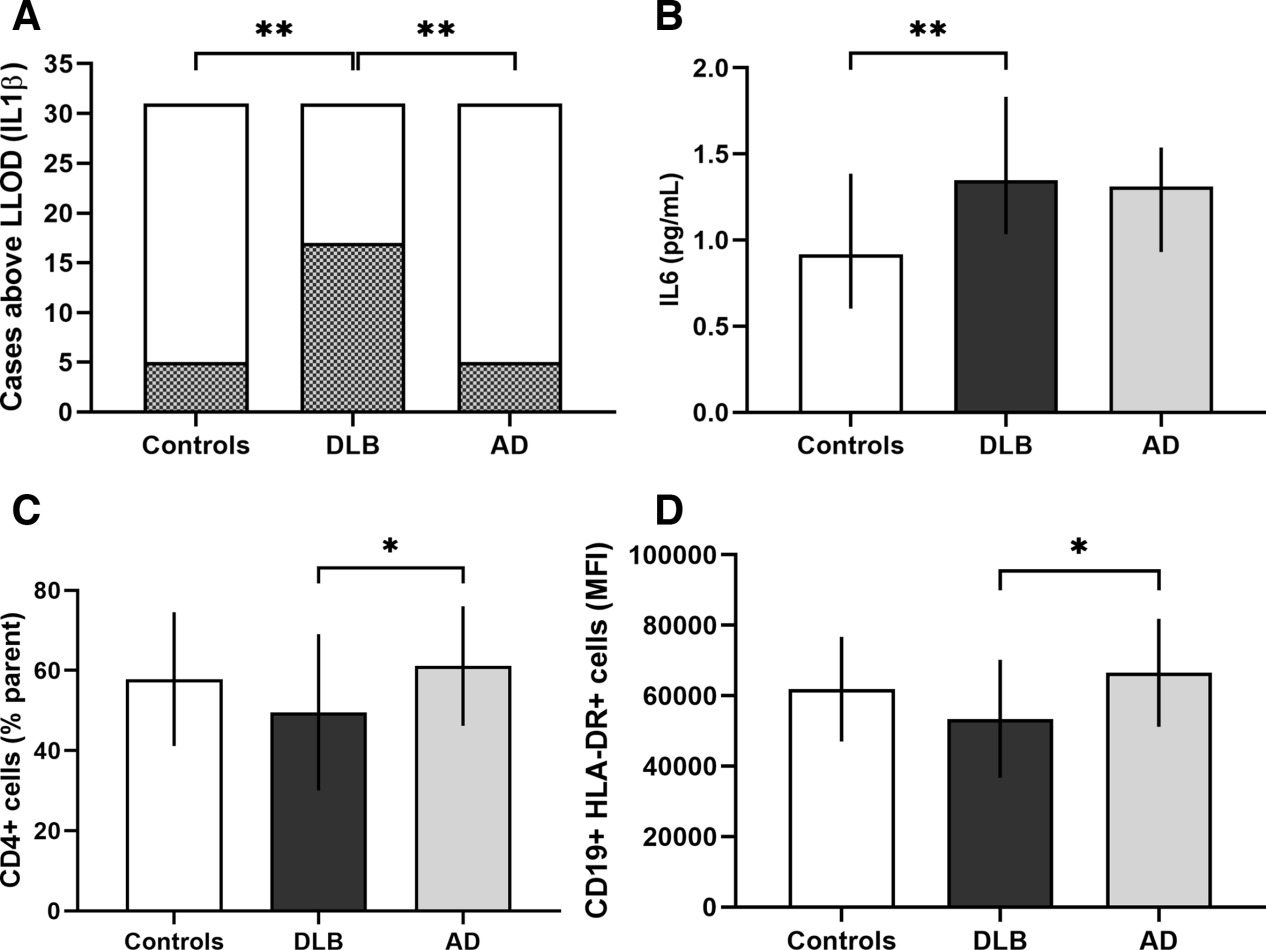
Significant cytokine and peripheral blood mononuclear cell results. Panel of graphs illustrating significant findings from cytokine and PBMC subset analysis. Top left: IL-1β, increased number of cases with serum concentrations above the lower limit of detection (shaded) in DLB compared with controls and AD. Top right: IL-6 (median with IQR), increased serum concentration in DLB compared with controls. Bottom left: CD4+ Tcells (mean with SD), decreased proportion of cells in DLB compared with AD. Bottom right: CD19+ HLA-DR+ activated B cell mean fluorescent intensity (mean with SD), decreased level of activation in DLB compared with AD. *p<0.05, **p<0.01. AD, Alzheimer’s disease; DLB, dementia with Lewy bodies; IL, interleukin; LLOD, lower limit of detection; MFI, mean fluorescent intensity.

Data from flow cytometry are summarised in table 2. The relative number of CD4+ helper T cells and the activation level of CD19+HLA-DR+ B cells were significantly different across groups. Post-hoc analysis showed a lower relative number of CD4+ helper T cells (p=0.043) and CD19+HLA-DR+MFI activated B cells (p=0.009) in DLB compared with AD. The difference in the relative number of CD4+ helper T cells between DLB and AD groups remained significant (mean difference 11.6 (95% CI 1.8–21.4), p=0.022) after correction for age and gender as possible confounders, as did the activation level of CD19+HLA-DR+ B cells (mean difference 13 164 (95% CI 4592–21736 standard units), p=0.003). Significant findings are illustrated in figure 1.

The relative number of CD8+ cytotoxic T cells appeared higher in DLB compared with controls and AD, but this did not meet statistical significance (p=0.053). Relative numbers of CD8+ terminal effector cells appeared elevated in DLB while naïve cytotoxic T cells were depleted compared with controls, but there were no significant group differences to justify post-hoc testing.

### Serum cytokine concentrations

IL-1β, IL-6, IL-10 and TNFα showed statistically significant differences between groups. Linear regression analysis showed serum IL-6 remained significantly higher in DLB than controls (p=0.015) after correction for age and gender. Differences in serum TNFα and IL-10 concentration were not statistically significant when linear regression was used to adjust for age and gender. IL-1β concentration was significantly different between groups (χ^2^(2)=15.030, p=0.001), with IL-1β-positive cases more prevalent in the DLB group (54.8%) compared with controls (16.1%, p=0.020), and AD (16.1%, p=0.003), following adjustment for age and gender (table 3). Significant findings are illustrated in figure 1.

**Table 3:** Serum cytokine concentrations

Cytokine	Controls (n=31)	DLB (n=31)	AD (n=31)	Test statistic P value
IL-1β	0.00 0.00–0.00	0.04^α,β^ 0.00–0.07	0.00 0.00–0.00	14.068* **0.001**
IL-2	0.11 0.00–0.30	0.25 0.15–0.33	0.24 0.13–0.35	4.162† 0.125
IL-4	0.09 0.011–0.19	0.17 0.13–0.21	0.16 0.04–0.21	3.929† 0.140
IL-6	0.92 0.60–1.35	1.35^α^ 1.03–1.83	1.31 0.94–1.51	**10.007**† **0.007**
IL-8	7.45 6.29–10.14	6.41 4.35–9.46	7.49 5.95–11.40	3.640† 0.162
IL-10	0.41 0.18–0.65	0.62 0.48–0.77	0.62 0.28–0.72	**6.307**† **0.043**
IL-12	0.21 0.13–0.39	0.29 0.20–0.43	0.29 0.06–0.41	1.270† 0.530
IL-13	1.46 0.00–2.32	2.35 1.43–2.80	2.17 0.57–2.77	4.623† 0.099
TNFα	1.51 1.32–1.91	1.86 1.51–2.74	1.82 1.47–2.38	**6.603**† **0.037**
IFN-γ	3.70 3.01–5.79	4.55 2.76–10.70	6.50 4.10–8.63	5.013† 0.082

Results are presented as median pg/mL with LQ-UQ below. Statistical tests are denoted with symbols.

Number of cases revalued to 0 as duplicate samples were below the assay lower limit of detection: IL-1β 66/93 (subsequently analysed as binary variable), IL-2 24/93, IL-4 18/93, IL-6 0/93, IL-8 0/93, IL-10 0/93, IL-12 19/93, IL-13 22/93, TNFα 0/93, IFN-γ 0/93.

Post-hoc results: α=significantly different from HC (p<0.05), β=significantly different from AD (p<0.05).

*Pearson χ^2^.

†Kruskal-Wallis.

IFN, interferon; IL, interleukin; TNF, tumour necrosis factor.

### Correlation of immune markers with clinical features of DLB

In DLB there were no significant associations between any PBMC subsets or cytokine concentrations with standardised rating scale scores representing severity of clinical features, including the MoCA (cognition), UPDRS (Parkinsonism), CAF (fluctuations), NPI (neuropsychological symptoms), CSDD (depression) and FCSRT-IR (recall).

## Discussion

In DLB, we have revealed diminished markers associated with humoral adaptive immunity, and a proinflammatory innate immune signature, supporting a unique peripheral immunophenotype. This was only possible by investigation of peripheral adaptive immunity using flow cytometry for the first time in this disease. Our findings have important implications in furthering our understanding of the aetiology of DLB.

Our study benefited from DLB and AD groups that were well matched for age, disease duration and educational attainment. Statistical correction was used to account for the significantly younger control group and non-significant differences in gender. All groups demonstrated low proportions of anti-inflammatory drug use. As anticipated, patients with DLB scored higher on scales associated with clinical features that characterise the disease for example, fluctuations (CAF) and motor features of Parkinsonism (UPDRS).

We demonstrated significantly lower relative numbers of CD4+ T cells in DLB compared with AD using flow cytometry. We found a trend for higher relative numbers of CD8+ T cells in DLB, but this was not statistically significant. Our findings are supported by previous PBMC analysis in both AD and PD showing reduced populations of helper T cells^21 22^ and unchanged relative numbers of cytotoxic T cells.^21 24 25^

**Figure 2:**
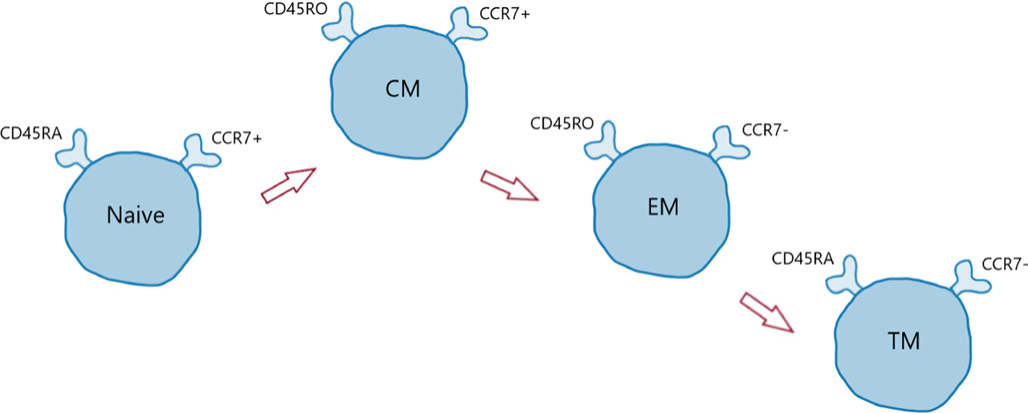
Profile of T cell differentiation. Illustration of changing expression of CD45RA and CCR7 during T cell differentiation. Naïve T cells express both CD45RA and CCR7. Central memory (CM) T cells lose CD45RA but still express CCR7. Effector memory (EM) T cells do not express CD45RA or CCR7. Terminally differentiated (TM) memory T cells re-express CD45RA but not CCR7. CD45RO denotes an absence of the CD45RA marker.

Further examination of T cell subsets and phenotype, using CD45RA and CCR7, allowed some preliminary insights into stage of maturity or differentiation. Figure 2 illustrates the CD45 and CCR7 profile found in T cell subsets, ranging from naïve to central memory, effector memory and finally to terminally differentiated memory cells (TM).^29^ We did not detect significant changes in helper T cell subsets in DLB and therefore our findings are likely driven by an overall decline in cell population, which has been shown in PD.^22^ Nevertheless, although group differences did not reach statistical significance, CD8+ cytotoxic T cell subset changes suggested a potential shift from a naïve towards a more terminally differentiated phenotype in DLB. Thus, rather than depletion of CD8+ T cells typically seen with ageing, the relative increase found in DLB, along with a more terminally differentiated phenotype, supports further studies to assess possible increased proliferation and senescence of cytotoxic T cells in DLB. T cells with a TM phenotype are known to be more commonly senescent, although confirmation of this phenotype in DLB would require further analysis, such as CD57 expression.^29^

We observed significantly decreased activation of HLA-DR+ B cells in DLB compared with AD. Reduced B cell activation could demonstrate a diminished level of humoral adaptive immunity in DLB, perhaps secondary to an impaired proliferative response to infection. This is supported by previous literature in PD that shows a reduction in B cells compared with controls.^22^

We report that IL-1β was detected more frequently in DLB compared with AD and controls, and elevated IL-6 concentration was found in DLB compared with controls. IL-1β is a major, acute phase, proinflammatory protein that is known to be a potent inducer of IL-6.^30^ IL-6 is a pivotal cytokine in the transition from innate to adaptive immunity, and is involved in T cell differentiation.^31^ However, in animal models IL-6 inhibits the production of IL-1 and TNFα, suggesting a role in regulating inflammation.^32^ Elevated serum IL-1β has been consistently reported in PD, PD dementia and AD.^19 33^ Our results extend this to DLB, but despite IL-1β polymorphisms being potential risk factors for AD and PD,^34 35^ this has not yet been shown in DLB, possibly as a result of limited genetic data to date. There may be a common role for IL-1β in the pathogenesis of all these neurodegenerative causes of dementia. In DLB its role may be associated with frequency of infections or falls associated with Parkinsonism, although we did not find this in our study. The role of serum IL-6 in DLB is more unclear due to its proinflammatory and anti-inflammatory properties. Elevation of this cytokine in our DLB group along with previously reported elevation in prodromal DLB,^16^ suggests that IL-6 may play a complex and varied role that changes with disease progression.

Two previous clinical studies have examined blood cytokine concentrations in DLB. One demonstrated unchanged cytokine concentrations in DLB, but increased levels of IL-1β, IL-2, IL-4 and IL-10 in MCI-DLB, the first three of which were lower in more severe disease.^16^ The other study showed increased IL-2 and IL-17, and reduced IL-8, in DLB.^17^ Taken with our results, these findings support altered innate immunity in DLB, possibly most prominent in early disease. However, there is a lack of reproducible findings relating to specific cytokines to date, possibly due to a lack of standardisation of methodology.^36^

We did not find significant associations between peripheral inflammation and the clinical features of DLB. Previous studies have reported elevated serum IL-10 with increased Parkinsonism,^16^ and increased IL-6 and TNFα with greater neuropsychiatric symptoms and worse cognition, respectively.^15^ Our study may not have been powered to detect significant associations using stricter significance thresholds due to multiple testing.

The strengths of this study include the large cohort of patients with AD and DLB identified using consensus diagnostic criteria and use of flow cytometry to examine PBMC subsets. Cryopreservation of PBMC allowed batch analysis using flow cytometry, a method that is widely used and although it can reduce cell viability it is comparable to using fresh whole blood,^37^ allowing us to minimise the effect of inter-assay variability. Limitations include the single time-point and lack of a PD group to allow direct comparison with previous studies. In addition, our control group was significantly younger than both dementia groups. Although we did statistically adjust for age as a potential confounder, it would have been optimal to match participants. Some methodological adjustments could be made in future research in this field. The flow cytometry panel could be enhanced to include markers of senescence (eg, CD57 or KLRG-1) or exhaustion (eg, PD1), providing more detail regarding the extent of T cell differentiation. PBMC data analysis in this study was partially limited by missing data for a small number of cases from each group, which could be mitigated in future by isolating more PBMC from each case. More detailed clinical information on factors such as diet and physical activity would have enabled us to correct for these potential confounding factors. Finally, quantification of PBMC subsets could be enhanced by the measurement of absolute cell counts, allowing calculation of cell ratios such as CD4:CD8.

Lymphocyte biomarkers could be used to aid early diagnosis of patients with AD but standardisation and validation of findings is not yet satisfactory.^38^ This may be due to differences in study sample size, study population demographics and differences in methodologies.^39^ The finding of a unique peripheral immune profile in DLB has the potential to direct future work examining mechanistic links between inflammation and disease progression. The identification of a reliable and accurate biomarker for DLB would allow earlier diagnosis, more tailored treatment and improve recruitment to interventional trials. Our study has shown a combination of decreased humoral adaptive immunity in DLB with possible shifts in cytotoxic T cell subsets towards a senescent phenotype. We propose that DLB is characterised by impaired proliferation or activation of B and helper T cells, with potentially chronically activated and quiescent cytotoxic T cells. It is plausible that these findings may be driven by the increased frequency that patients in DLB encounter infections,^40^ although the direction of effect is unable to be established in a cross-sectional study.

In conclusion, we have demonstrated hypothesis-generating alterations to adaptive and innate immunity in DLB. Further work is warranted to explore the immunophenotype of DLB across the spectrum of disease severity. This should include large-scale genetic work to examine the possibility as to whether polymorphisms in genes associated with inflammation are implicated in DLB. Longitudinal analysis of peripheral inflammation in DLB, from prodromal to terminal disease, will enhance our understanding of the aetiology of DLB and may identify diagnostic biomarkers and novel targets for intervention.
